# Effect of hydroxytyrosol, Moringa, and spirulina on the physicochemical properties and nutritional characteristics of gluten‐free brownies

**DOI:** 10.1002/fsn3.3778

**Published:** 2023-10-18

**Authors:** Rocío Peñalver, Lorena Martínez‐Zamora, José Manuel Lorenzo, Gaspar Ros, Gema Nieto Martínez

**Affiliations:** ^1^ Department of Food Technology, Food Science and Nutrition, Faculty of Veterinary Sciences, Regional Campus of International Excellence “Campus Mare Nostrum” University of Murcia Murcia Spain; ^2^ Centro Tecnológico de la Carne de Galicia Parque Tecnolóxico de Galicia Ourense Spain; ^3^ Área de Tecnología de los Alimentos, Facultad de Ciencias de Ourense Universidad de Vigo Ourense Spain

**Keywords:** antioxidant, fiber, functional food, gluten‐free brownies, hydroxytyrosol, Moringa, spirulina

## Abstract

Brownies, enriched with fiber and *Moringa oleifera*, hydroxytyrosol (HXT), and spirulina (encapsulated and nonencapsulated), and dietary fiber using psyllium were elaborated. For that, a commercial control (CTRL) and an experimental control (CTRL) (without antioxidants) were compared. Color, nutritional composition, pH, antioxidant capacity, total phenolic compounds, as well as sensory properties were evaluated. The results showed the brownies developed with psyllium and the different extracts increased total dietary fiber compared to CTRL Commercial and CTRL, with E‐spirulina (14.93 g/100 g) and Moringa (11.91 g/100 g) being the most prominent samples. However, with regard to soluble fiber, the highest content of NE‐spirulina and the lowest content of HXT were observed. In addition, brownies enriched with extracts showed higher antioxidant capacity and higher phenolic compounds than controls, with HXT standing out at 299.25 μM Trolox/g in ABTS, 1470.08 μM Trolox/g in DPPH, and 18.15 μM Trolox/g in FRAP. However, a high level of phenolic compounds was found in E‐Spirulina (604.71 mg/L). In reference to fatty acids, monounsaturated fatty acids (MUFA) (70%) were the predominant fatty acids, followed by polyunsaturated fatty acids (PUFA) (19%) and saturated fatty acids (SFA) (10%). Glutamic acid and asparagine were the predominant amino acids. As for mineral content, N‐spirulina and E‐spirulina were the brownies with the highest iron bioaccessibility; Si, Na, B, Al, P, Fe, Zn, Bi, Ca, Cu, Mg, Mn, Rb, S, and Sr being the most abundant elements in the brownies of this study. In addition, the HXT and Moringa samples scored higher in overall acceptability compared to the controls. The findings suggest that the incorporation of psyllium, quinoa flour, and antioxidant extracts in brownies could be a viable approach to produce a healthy brownie enriched with fiber, antioxidants and, therefore, considering the nutritional, physicochemical, and organoleptic characteristics, HTX is the ideal compound to enrich bakery products.

## Graphical abstract

Development of gluten‐free brownie enriched with Moringa, hydroxytyrosol, spirulina, and fiber. HTX is the ideal compound to enrich bakery products.

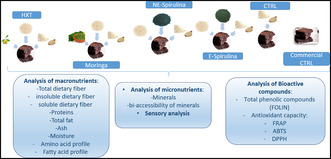

## 1 INTRODUCTION

The global bakery market is experiencing strong growth due to the increasing popularity of customizable cakes and the growing demand for innovative food products (European Regional Development Fund, 2021). Although value of the global bakery products industry has reached an estimated value of USD 350 billion in 2020, and is expected to grow by more than USD 100 billion in the next 5 years, the World Health Organization recommended in 2004 that the industry reduce the amount of energy, saturated fats, trans‐fats, sodium, and added sugars contained in these kind of products (Hughes, 2006).The development of fortified bakery products aims to improve the nutritional status of patients while modifying other product characteristics, such as digestibility, dough stability, antioxidant capacity, and shelf life, among others (Milla et al., 2021).

In particular, brownies are chocolate‐flavored baked goods in a square or rectangular shape. They are commonly eaten around the world, although they originated in the United States, and are made with flour, fat, sugar, eggs, and chocolate. The high concentrations of polyphenols in cocoa are attributed to the formation of proanthocyanidins, consisting of catechin monomers and its isomer epicatechin (Brewer, 2011). Proanthocyanidins have been shown to ameliorate abnormalities in complex I of the mitochondrial respiratory chain induced by rotenone, an inhibitory compound used as a pesticide and herbicide, in a dopaminergic cell line. Proanthocyanins, therefore, emerge as a potent neuroprotective potential against degenerative diseases (Gu et al., 2011). In addition, the most antioxidant compound known, hydroxytyrosol (HXT) is a phenolic compound extracted as a by‐product of the olive tree. The number of biological actions performed by HXT is enormous, including anti‐inflammatory effects, antithrombotic, hypocholesterolemic activities, negative regulation of the immune response, and protection of various human cell types from oxidative damage induced by reactive oxygen species, resulting in a lower frequency of cell death and a significant increase in cell half‐life. This antioxidant activity is justified by its chemical structure: A phenolic ring consisting of a catechol group and three hydroxyl groups, the combination of which is the main reason for its preservative action (Bertelli et al., 2020; Cabrerizo et al., 2013; Martínez et al., 2018; Robles‐Almazan et al., 2018).

Another interesting antioxidant is *Moringa oleifera*, used for centuries as a medicinal plant for its wide range of nutritional and bioactive compounds, it has a potent anti‐inflammatory effect that is justified by its content of flavonoids, alkaloids, tannins, and glycosides. Due to its extensive nutritional and medicinal properties, the leaves of this plant have been selected as a source for obtaining a potential extract to be included in the preparation of functional and medicinal foods. In fact, many authors have experimented with incorporation mainly in products such as biscuits, cakes, brownies, meats, juices, and sandwiches (Milla et al., 2021).

Seaweed is an excellent source of macro‐ and micronutrients, including a high proportion of proteins, vitamins, minerals, and dietary fiber, particularly rich in the soluble fraction (Soni et al., 2017). Studies on the antioxidant effects of spirulina in humans have shown positive effects on health and physical performance, which is thought to be due to the promotion of fat oxidation (Soni et al., 2017).

Psyllium is a small annual herb that is part of the Plantaginaceae family and consists of hydrophilic polysaccharides that are not digestible by human intestinal enzymes. One study found that psyllium supplementation led to marked changes in the microbiota of constipated patients compared to healthy patients, resulting in an increase in the relative amount of water in the stool of constipated patients and consequently an alleviation of pain (Jalanka et al., 2019).

Studies using quinoa (*Chenopodium quinoa*) as a nutritional supplement in the diet have reported gastroprotective activity. These effects are mostly attributed to arabinose and the arabinose‐rich pectic polysaccharides that constitute dietary fiber (Nascimento et al., 2014). NASA has classified quinoa as a crop with excellent nutritional characteristics for use in long‐lasting space missions due to its high protein content and unique composition of sulfur amino acids and lysine (Nascimento et al., 2014).

In this sense, the main objective of the work was to manufacture different functional brownies enriched in fiber and antioxidant compounds that can be incorporated into the diet of population groups at risk of chronic diseases, based on their characteristic nutritional deficiencies. To achieve this objective, natural extracts of *Moringa oleifera*, hydroxytyrosol, and spirulina were characterized for their antioxidant, anti‐inflammatory, and neuroprotective properties. These ingredients were used in new brownie formulations enriched also in fiber by the use of quinoa flour and psyllium

## 2 MATERIALS AND METHODS

### 2.1 Plant extracts

Antioxidant plant extracts used in the present experiment were as follows: *Moringa oleifera* powder (100% dried moringa leaves from KeyPharm Laboratories [Oostkamp, Belgium]), hydroxytyrosol from olives leaves (supplied by Nutrafur S.A. [Alcantarilla, Murcia, Spain]), and spirulina supplied by Porto‐Muiños S.L. (A Coruña, Spain). Encapsulation method was developed according to Mohammadi et al., 2021 with some modifications. For that, 15 g of spirulina powder were homogenized in a solution of Maltodextrin: Arabic gum (60:40). After stirring overnight at room temperature, the mix was spray‐dried using a Mini Spray Dryer (Büchi Labortechnik, Flawil, Switzerland). The inlet and outlet air temperature were 140°C and 70°C, respectively (aspirator and feed 80% and 25%, respectively; flow rate was 55 m^3^ h^−1^). Dried powders were stored in vacuum bags at room temperature.

### 2.2 Brownie formulation

A brownie enriched in antioxidant compounds such as organic HXT, *Moringa oleifera*, and spirulina was made with special richness in fiber of quinoa flour and psyllium. The creation of a new potentially functional brownie was developed by introducing psyllium and an antioxidant compound in each of the samples except for the control, in which neither psyllium nor any additional extract was used. The quinoa flour was used in all the studied formulas for its high content of fiber, protein, and minerals such as calcium, iron, manganese, magnesium, copper, and potassium compared to other conventional flours. Aside from that, a commercial control was used, for which “Mix Brownies with Belgian chocolate” from Schär (Burgstall, Italy) was chosen. In order to determine the appropriate concentration of each of the compounds added to the original recipe, a sensory analysis was carried out by trained panelists with the aim of achieving organoleptic acceptability prior to the physicochemical analysis of the samples. Finally, the concentrations selected for each of the samples were as follows: 1200 ppm of Moringa, 550 ppm of HXT, 2000 ppm of encapsulated spirulina, and 1500 ppm of nonencapsulated spirulina. On this basis, four different brownies were produced, differing from each other in the antioxidant compound added, as shown in Table 1.

**TABLE 1:** List of ingredients (g) used for the reformulation of the five studied brownies.

Ingredients	CTRL Commercial	CTRL	Moringa	HXT	E‐Spirulina	NE‐Spirulina
Commercial mix	500	–	–	–	–	–
Quinoa flour	–	170	120	120	120	120
Psyllium	–	–	50	50	50	50
EVOO	250	250	250	250	250	250
Sugar	–	180	180	180	180	180
Baking powder	–	25	25	25	25	25
Cocoa 70%	–	65	65	65	65	65
Ovoproduct	325	325	325	325	325	325
Antioxidant						
Moringa			0.8			
HXT				0.4		
E‐Spirulina					1.4	
NE‐Spirulina						1.0
Visual appearance of the extracts			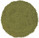	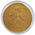	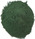	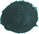
Visual appearance of the brownies	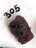	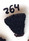	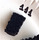	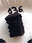	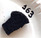	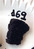

Abbreviations: E, Encapsulated; EVOO, Extra virgin olive oil; NE, Nonencapsulated. *Note*: Commercial mix (Schär): Whole cane sugar, maize starch, maize flour, cocoa powder, chocolate chips 7.4% (sugar, cocoa mass 33%, cocoa butter, emulsifier: soya lecithin, natural flavorings), rice flour, lentil flour, modified tapioca starch, and thickener: tara gum.

### 2.3 Product characterization

#### 2.3.1 Analysis of macronutrients

Moisture, dry matter, fat (Soxhlet), protein (Kjeldahl), dietetic fiber, and carbohydrates analyses were carried out according to standardized methods (AOAC, 1995). The main macronutrients were analyzed and the total energy was calculated.

The fatty acid profile was analyzed following the method described by Domínguez et al. (2015). For that, extracted samples (Domínguez et al., 2015) were analyzed in a gas chromatograph (GC‐Agilent 6890 N; Agilent Technologies Spain, S.L., Madrid, Spain) with a flame ionization detector for the separation and quantification of fatty acids. The chromatographic conditions were as follows: initial column temperature 120°C maintained for 5 min, programmed to increase at a rate of 5°C min up to 200°C, maintained for 2 min, then at 1°C min^−1^ up to 230°C, and maintained for 3 min. The injector and detector were maintained at 260°C and 280°C, respectively. He was used as carrier gas at a constant flow rate of 1.1 mL min^−1^, and the column head pressure was set at 35.56 psi. The split ratio was 1:50 and 1 μL of solution was injected. Individual FAMEs were identified by comparing their retention times with those of authentic commercial standards (Supelco 37 component FAME Mix, Sigma‐Aldrich, Madrid, Spain). Data were expressed as mg 100 g^−1^ of sample.

The amino acid profile was analyzed following the method described by Shahidi and Synowiecki (1993) with some modifications. For that, 0.1 g of brownie was mixed with 5 mL of 6 N HCl in a sealed glass tube for 24 h at 110°C. After that, the solution was then diluted with 200 mL of distilled water and filtered through a 0.45 μm. Amino acids were derivatized using 6‐aminoquinolyl‐N‐hydroxysuccinimidyl carbamate (Waters AccQ‐Fluor reagent kit) and determined by HPLC as previously described by Domínguez et al. (2015). Amino acids were identified using an amino acid standard (Amino Acid Standard H, Thermo, Rockford, USA) and their content was expressed as mg 100 g^−1^.

#### 2.3.2 Analysis of micronutrients

The method of mineral determination consists of the measurement of atomic emission using inductively coupled plasma mass spectrometry (ICP‐MS) (Thermo electron X7 inductively coupled plasma mass spectrometry, model X series, United Kingdom) as previously described by Martínez, Ros, and Nieto (2020). ICP‐MS operating conditions were described by Peñalver et al. (2022) as following: nebulizer gas flow, 0.91 L min^−1^; radio frequency (RF) 1200 W; lens voltage 1.6 V; cool gas 13.0 L/min; and auxiliary gas 0.70 L min^−1^. The mineral content of the samples was assessed and the digested samples, which were in vitro digested followed the method described by Peñalver et al. (2022).

#### 2.3.3 pH and color

The pH of brownies samples was measured in triplicate using Crison GLP21 equipment (Crison Instruments S.A., Barcelona, Spain). For that, 1 g of sample were mixed with 5 mL of water and homogenized. Color was assessed using a Konika Minolta CR 400 chromameter (Minolta Camera Co., Osaka, Japan). Lightness (L*), coordinates a* and b*, Chroma, and hue° angle were measured according to the CIELab system.

### 2.4 Total phenolic content

The total phenolic content was spectrophotometrically assessed using the Folin–Ciocalteu reagent and gallic acid as the standard following the method previously described by Singleton and Rossi (1965). The total phenolic content was expressed as mg gallic acid equivalents (GAE) per g sample.

### 2.5 Total antioxidant capacity

The antioxidant activity of the brownies’ samples was spectrophotometrically assessed following different methods: Ferric Reducing Antioxidant Power (FRAP) (Benzie & Strain, 1996), and the radical cation scavenging capacity against ABTS+ (2,2′‐azino‐bis(3‐ethylbenzothiazoline‐6‐sulphonic acid)) (Re et al., 1999) and DPPH (2,2‐difenil‐1‐picrilhidracilo) (Brand‐Williams et al., 1995)‐free radicals. The total antioxidant capacity was expressed as mg Trolox equivalents (TE) per g sample.

### 2.6 Sensory analysis

The sensory analysis was carried out by means of a hedonistic tasting in which 25 trained panelists aged between 20 and 59 years participated. The analysis comprised the evaluation of the six different types of brownies elaborated as described in Section 2.2. It was carried out in insulated booths in accordance with ISO‐8586, 2014. The booths are separated from each other by high, wide partitions that isolate the participants of the study, thus preventing the possible influence of the perception of the other panelists. The parameters of appearance, color, aroma, texture, juiciness, taste, purchase intention, and overall acceptance were evaluated on a scale from 1 to 5, 1 being the worst (“I don't like it at all”) and 5 the best (“I like it a lot”).

### 2.7 Statistical analysis

All analyses were carried out in triplicate. A descriptive analysis and an analysis of variance (ANOVA) were performed using SPSS software (vs 27.0, IBM, Armonk, NY, USA). Statistical significance was set at *p* ≤ .05 and Tukey's multiple range test was used to establish and separate means.

## 3 RESULTS AND DISCUSSION

### 3.1 Proximal composition

The proximal composition of reformulated brownies is shown in Table 2. As observed, moisture and ashes contents were mostly similar in all the studied samples. Nevertheless, we can observe important reductions of fiber and protein contents in CTRL commercial sample in comparison with the rest of the samples. Specifically, the fiber content was slightly higher in CTRL sample, although it was still lower than the half of the total fiber of Moringa, HXT, E‐Spirulina, and NE‐Spirulina samples. Related to this fact, the carbohydrates represent more than the 22% of the total formulas, while the CTRL commercial had more than 33%, from which the 30% of that were free sugars, compared to the rest of formulas, which presented the half (15%) of simple carbohydrates if we consider the total fiber values.

**TABLE 2:** Proximal composition (g 100 g^−1^) of new formulas of brownies.

Brownie formula		Moisture	Ashes	Carbohydrates	Soluble fiber	Insoluble fiber	Total fiber	Fat	Proteins
CTRL Commercial	Mean	42.26	1.87	33.16	2.47	1.36	3.83	17.57	5.13
	SD	1.44	0.02	1.66	0.10	0.20	0.19	0.40	0.08
		CD	B	A	C	E	E	C	E
CTRL	Mean	42.89	2.33	23.09	0.10	5.31	5.41	23.66	8.03
	SD	3.05	0.61	1.15	0.17	0.81	0.27	1.73	0.10
		BC	AB	C	B	D	D	A	A
Moringa	Mean	39.85	2.47	32.84	2.38	9.53	11.91	17.52	7.42
	SD	0.15	0.06	1.64	0.28	0.62	0.60	0.46	0.21
		D	A	A	B	C	C	C	B
HXT	Mean	43.76	2.41	28.81	1.15	11.53	12.67	18.69	6.34
	SD	0.83	0.01	1.44	0.21	0.47	0.63	0.02	0.18
		BC	AB	B	C	B	C	C	D
E‐Spirulina	Mean	45.91	2.42	22.10	2.31	12.62	14.93	22.81	6.76
	SD	1.44	0.17	0.11	0.32	1.44	0.75	0.04	0.21
		AB	A	C	B	A	A	AB	C
NE‐Spirulina	Mean	47.08	2.40	22.34	3.56	9.96	13.52	21.36	6.83
	SD	0.61	0.25	1.12	0.06	0.67	0.68	1.19	0.04
		A	AB	C	A	C	B	B	C

*Note*: Different capital letters denote significant differences among reformulated brownies (*p* < .05).

CTRL brownies have higher protein content than the CTRL commercial brownies. This fact can be attributed to the replacement of a commercial flour with quinoa flour, as it has been shown that its use leads to an increase in protein content, as well as a unique composition in the sulfur amino acids lysine and sulfur (Nascimento et al., 2014). Of the brownies enriched with different types of antioxidant compounds, the value of *Moringa oleifera* (Moringa brownie) stands out, which has a higher protein content, which is supported by previous results obtained (Milla et al., 2021) highlighting the essential amino acid content of the leaf of this herbaceous species.

Similar to protein content, CTRL brownies shows higher fiber values than the CTRL commercial due to the use of quinoa flour, which is consistent with the baseline hypothesis and with previous studies in which quinoa was used for the purpose of increasing total dietary fiber (Kurek et al., 2018). This increase is due to the insoluble fraction, which is consistent with previous results where the proportion of soluble dietary fiber was only 13.5% of total dietary fiber in quinoa, as cooking altered the soluble fraction while insoluble fiber remained unchanged (Arendt & Bello, 2008).

The results show a marked increase in total dietary fiber in products supplemented with quinoa and psyllium meal (Moringa, HTX, E‐Spirulina, and NE‐Spirulina), largely due to insoluble fiber. The elevation of insoluble dietary fiber in brownies supplemented with psyllium contrasts with previous results as this hydrophilic polysaccharide is used commercially as a source of soluble fiber (Campbell & Fahey, 1997).

In a similar study in which breads fortified with fiber and different antioxidants were developed, it was observed that supplementation with 5% psyllium led to an increase in the moisture content of the breads (around 43% moisture) while at the same time causing a reduction in the density of the breads (Park et al., 1997).

### 3.2 Characterization pH and color

Table 3 shows the obtained results of color and pH measurements. Regarding the pH values obtained, it is known that the optimal yeast activity is produced at pH range between 6 and 8. In this sense, it is remarkable that although all the samples presented values near neutrality, there is a notable increase in pH in the experimental brownies compared to the CTRL commercial, which can be due to the type of flour and the list of ingredients or additives used in the commercial mix. Therefore, while the neutral pH of the CTRL commercial must be related to its list of ingredients (whole cane sugar, maize starch, maize flour, cocoa powder, chocolate chips 7.4% (sugar, cocoa mass 33%, and cocoa butter), emulsifier: soya lecithin, natural flavorings, rice flour, lentil flour, modified tapioca starch, and thickener: tara gum), the pH of the reformulated samples can justify by the pH of quinoa flour (around 6, according data obtained by Brito et al., 2015; Franco et al., 2021; Xing et al., 2023), which is increased by the addition of sugar to the formula by decreasing the concentration of positively charged ions in the dough.

**TABLE 3:** pH and Color of reformulated brownies.

Brownie formula		pH	L*	a*	b*	Chroma	Hue°
CTRL commercial	Mean	7.08	29.2	11.82	11.51	16.5	30.41
	SD	0.07	1.46	0.59	0.57	0.82	1.52
		E		A	A	A	
CTRL	Mean	8.37	26.32	8.07	5.05	9.6	30.41
	SD	0.08	1.32	0.40	0.25	0.48	1.52
		D		AB	B	B	
Moringa	Mean	8.55	24.06	5.11	2.61	5.75	26.06
	SD	0.10	1.20	0.25	0.13	0.29	1.30
		AB		B	B	B	
HXT	Mean	8.64	24.18	5.29	2.86	6.05	26.11
	SD	0.12	1.21	0.26	0.14	0.30	1.31
		A		B	B	B	
E‐Spirulina	Mean	8.44	25.09	4.12	2.14	4.73	20.99
	SD	0.06	1.26	0.21	0.11	0.24	1.05
		BC		B	B	B	
NE‐Spirulina	Mean	8.39	24.03	4.47	3.12	5.29	23.49
	SD	0.03	1.20	0.22	0.16	0.26	1.17
		C		B	B	B	

*Note*: Different capital letters denote significant differences among reformulated brownies (*p* < .05).

Also, as shown in Table 3, the sample with the highest a* and b* values is the CTRL commercial, followed by CTRL and the brownies enriched with an antioxidant extracts, among which no major changes are noticeable. This pattern is repeated for Chroma parameter (intensity of color), although no significant differences were found in relation to brightness (L*) and tone (Hue angle).

A study on the effect of the addition of sugars on the colorimetry of chocolate found that the luminosity of the samples decreased as the proportion of sugars decreased, regardless of whether they were refined or not, which would explain the high values of this parameter in the commercial control, whose percentage of carbohydrates exceeds 70% of the nutritional value (Hernández & Cerón, 2016). This same study revealed that the a and b coordinates slightly increased their values due to the presence of unrefined sugars such as panel a, so this could explain the elevation of these parameters with respect to the brownies made experimentally. In this sense, also the increases shown in chromaticity of the color in CTRL commercial can due to the higher proportion of cocoa butter and possible cocoa with lower purity included in the commercial mix used in such formula in comparison with the 6% of cocoa 70% used in our reformulated samples, which apparently shows a darker color as shown in Table 1.

### 3.3 Fatty acids profile

**FIGURE 1:**
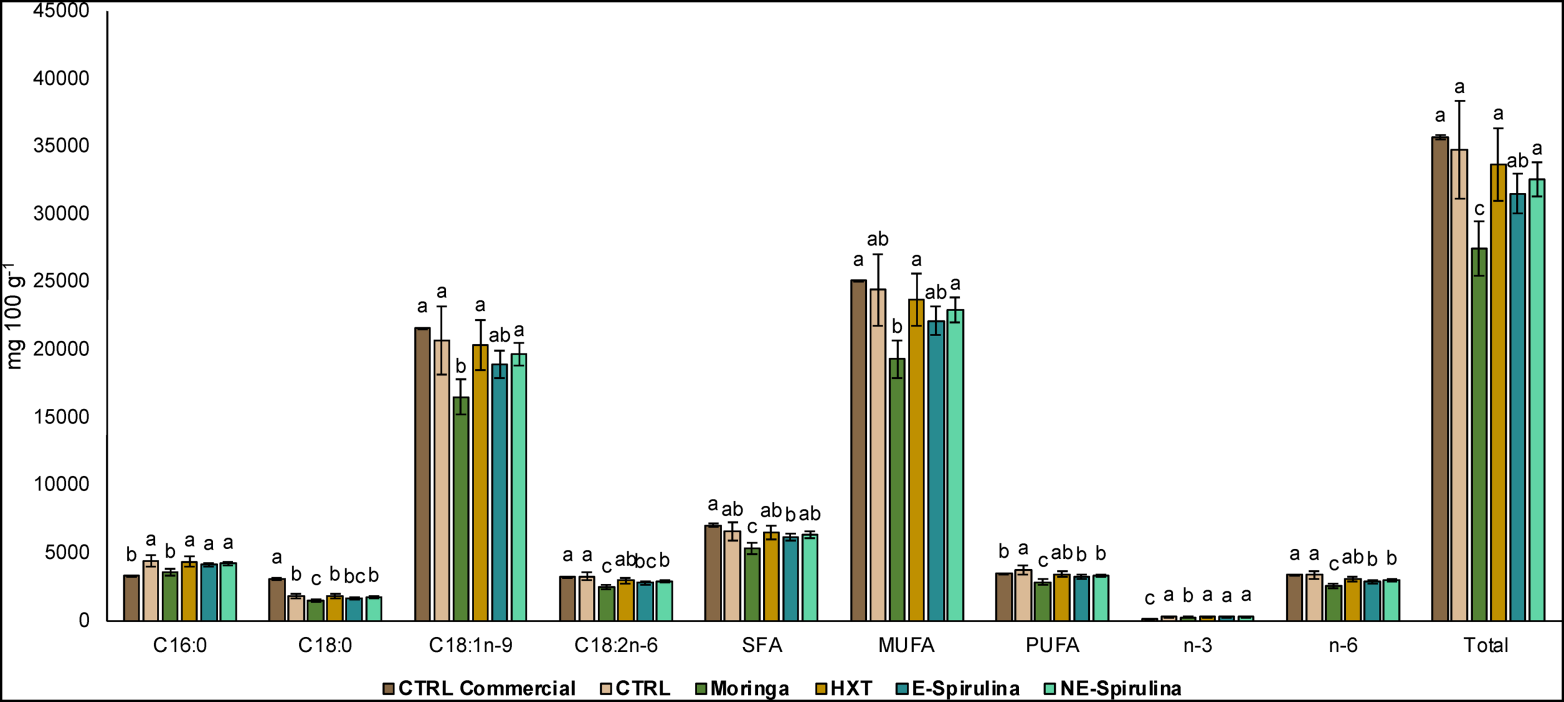
Main fatty acids and total profile (mg 100 g^−1^) of studied formulas of brownies. Different letters denote significant differences among reformulated brownies (*p* < .05).

As shown in Table 4 and Figure 1, a total of 33 fatty acids conformed the profile of fatty acids of the studied brownies. These were in order of elution: butyric acid (C4:0), caproic acid (C6:0), caprylic acid (C8:0), capric acid (C10:0), lauric acid (C12:0), myristic acid (C14:0), myristoleic acid (C14:1n‐5), pentadecylic acid (C15:0), cis‐10‐pentadecenoic acid (C15:1n‐5), palmitic (C16:0), palmitoleic acid (C16:1n‐7), margaric acid (C17:0), stearic acid (C18:0), elaidic acid (9 t‐C18:1), oleic acid (C18:1n‐9), vaccenic acid (C18:1n‐7), linoleic (C18:2n‐6), calendic acid (C18:3n‐6), α‐linolenic (C18:3n‐3 (ALA)), conjugated linoleic acid (9c,11 t‐C18:2), arachidic acid (C20:0), gondoic acid (C20:1n‐9), dihomo‐linoleic (C20:2n‐6), heneicosylic acid (C21:0), dihomo‐γ‐linolenic (C20:3n‐6), arachidonic (C20:4n‐6), dihomo‐α‐linolenic (C20:3n‐3), behenic acid (C22:0), erucic acid (C22:1n‐9), all cis‐13,16‐docosadienoic acid (C22:2n‐6), tricosylic acid (C23:0), lignoceric acid (C24:0), and docosahexaenoic (C22:6n‐3 (DHA)).

**TABLE 4:** Profile of minor fatty acids (mg 100 g^−1^) of studied formulas of brownies.

	CTRL commercial	CTRL	Moringa	HXT	E‐Spirulina	NE‐Spirulina
C4:0	30.1 ± 0.8AB	28.6 ± 0.1AB	23.0 ± 7.3B	28.9 ± 0.7AB	34.9 ± 1.8A	32.0 ± 6.0A
C6:0	18.2 ± 0.6A	0.0 ± 0.0B	0.0 ± 0.0B	0.0 ± 0.0B	0.0 ± 0.0B	0.0 ± 0.0B
C8:0	15.9 ± 0.3AB	15.2 ± 0.1AB	12.4 ± 3.8B	15.3 ± 0.3AB	18.5 ± 1.2A	16.8 ± 3.0A
C:10	16.0 ± 0.3A	10.5 ± 0.0 BC	8.6 ± 2.7C	10.5 ± 0.3 BC	12.7 ± 0.7B	11.7 ± 2.1B
C12:0	16.2 ± 0.5A	8.5 ± 0.2 BC	6.7 ± 1.9C	8.3 ± 0.1 BC	9.8 ± 0.6B	9.0 ± 1.4B
C14:0	56.9 ± 1.0A	23.5 ± 1.1B	20.1 ± 2.4C	24.7 ± 1.3B	24.9 ± 1.1B	24.1 ± 1.4B
C14:1n‐5	6.5 ± 0.2A	4.3 ± 0.1 BC	3.8 ± 0.8C	4.6 ± 0.1 BC	5.1 ± 0.2B	4.9 ± 0.7B
C15:0	11.8 ± 0.3A	8.4 ± 0.1B	6.2 ± 0.7D	7.5 ± 0.3C	7.7 ± 0.1 BC	7.7 ± 0.5C
C15:1n‐5	3.5 ± 0.1B	4.5 ± 0.1A	3.4 ± 0.6B	4.1 ± 0.1A	4.3 ± 0.1A	4.3 ± 0.4A
C16:1n‐7	122.4 ± 0.6C	308.5 ± 32.9A	242.1 ± 18.8B	300.5 ± 25.3A	283.6 ± 12.1A	287.9 ± 11.5A
C17:0	28.4 ± 0.5 BC	33.4 ± 2.9A	26.5 ± 2.1C	32.5 ± 2.2A	31.0 ± 1.5AB	31.7 ± 1.4AB
9 t‐C18:1	32.1 ± 0.1A	14.1 ± 1.0 BC	13.8 ± 3.4C	15.6 ± 0.6 BC	17.4 ± 0.7B	17.0 ± 1.9 BC
C18:1n‐7	260.1 ± 2.4C	621.0 ± 69.2A	482.9 ± 39.2B	601.9 ± 52.5A	572.4 ± 33.6A	597.4 ± 35.5A
C18:3n‐6	7.1 ± 0.0CD	7.7 ± 0.6C	6.5 ± 1.1D	7.6 ± 0.0C	11.7 ± 0.0A	9.0 ± 0.7B
C18:3n‐3	50.9 ± 0.5D	241.8 ± 24.6A	178.3 ± 12.2	216.0 ± 17.2AB	202.9 ± 9.1 BC	208.3 ± 6.7B
9c.11 t‐C18:2 (CLA)	42.6 ± 1.5 BC	46.1 ± 0.3ABC	37.8 ± 11.2C	46.8 ± 0.9ABC	56.0 ± 3.5A	51.6 ± 9.6AB
C20:0	134.1 ± 2.9A	141.0 ± 16.8A	111.1 ± 9.9B	137.2 ± 11.3A	127.7 ± 6.5AB	133.2 ± 6.4A
C20:1n‐9	70.0 ± 0.0B	89.1 ± 8.2A	72.2 ± 5.5B	85.3 ± 3.0A	81.9 ± 4.6A	85.6 ± 3.3A
C20:2n‐6	13.0 ± 0.2A	14.1 ± 0.5A	10.3 ± 1.4B	12.4 ± 0.3A	13.1 ± 0.4A	12.6 ± 1.4A
C21:0	5.4 ± 0.1B	8.2 ± 0.8A	6.3 ± 1.1B	8.0 ± 0.4A	8.7 ± 0.5A	8.5 ± 1.2A
C20:3n‐6	8.5 ± 0.1A	8.5 ± 0.1A	6.9 ± 1.2B	8.2 ± 0.3A	8.1 ± 0.1A	7.7 ± 0.1AB
C20:4n‐6	74.6 ± 1.0B	82.6 ± 1.3A	74.3 ± 5.5B	85.2 ± 0.7A	82.1 ± 2.1A	84.7 ± 0.5A
C20:3n‐3	0.0 ± 0.0C	6.3 ± 0.1A	5.1 ± 1.2B	5.2 ± 0.2B	6.7 ± 0.3A	6.0 ± 0.7AB
C22:0	232.3 ± 0.8A	49.4 ± 4.9B	39.1 ± 4.2C	47.8 ± 2.0B	46.6 ± 2.1B	47.6 ± 3.5B
C22:1n‐9	2.19 ± 0.2C	14.7 ± 1.0AB	13.7 ± 1.4B	15.8 ± 0.6A	13.6 ± 0.1B	14.8 ± 0.7AB
C22:2n‐6	0.00 ± 0.0D	4.3 ± 0.3 BC	3.5 ± 0.9C	4.5 ± 0.2ABC	5.0 ± 0.1AB	5.6 ± 1.0A
C23:0	12.4 ± 0.3A	10.7 ± 1.2AB	8.7 ± 1.8C	10.5 ± 0.3 BC	11.1 ± 0.5AB	11.0 ± 0.8AB
C24:0	86.0 ± 0.2A	27.1 ± 3.7B	22.1 ± 3.5B	26.4 ± 2.6B	25.3 ± 2.1B	25.4 ± 2.0B
C22:6n‐3 (DHA)	34.2 ± 1.1C	47.9 ± 1.4AB	44.9 ± 3.3B	50.7 ± 0.4A	49.5 ± 0.7A	49.6 ± 0.4A

*Note*: Different capital letters denote significant differences among reformulated brownies (*p* < .05).

From a general point of view, the monounsaturated fatty acids represented the 70% of the total fat content, while the polyunsaturated fatty acids were the 19% and the saturated fatty acids the 10% (Figure 1). From the polyunsaturated fatty acids, the n‐6 was the 13%, being mainly linoleic (C18:2n‐6), the n‐3 was the 1.1%, being ALA (C18:3n‐3), EPA (C20:3n‐3), and DHA (C22:6n‐3), but the major fatty acid found was a monounsaturated fatty acid, oleic acid (C18:1n‐9), which was found in 85%.

In this sense, in Figure 1, the major fatty acids found in the brownies were palmitic (C16:0), stearic (C18:0), oleic (C18:1n‐9), and linoleic (C18:2n‐6). It is obvious that oleic acid was the major fatty acid found in the studied brownies when the brownies were made of EVOO in a 23% (20 g per 100 g of sample, approximately). As previously said, other minor compounds were identified and quantified and obtained values are shown in Table 4. In this sense, after the major compounds, the reformulated brownies are also sources of palmitoleic acid (C16:1n‐7), vaccenic acid (C18:1n‐7), ALA (C18:3n‐3), and arachidic acid (C20:0). The rest of the compounds were found in lower amounts (<0.1 g per 100 g of brownie).

Although the related bibliography is scarce, similar bakery products have obtained comparable results. For instance, palmitic, oleic, and lauric acid were also the main fatty acids found in brownies made of sweet potato flour with the addition of black cumin oil at different concentrations as main antioxidant agent (Ligarnasari et al., 2018). This high percentage of fat (around 38%) is also similar to this obtained by Raza et al. (2021) when elaborated their brownies enriched in mano leaves powder extract. Other authors with other kind of flours as Pedada Mangrove (Sumartini et al., 2021) also reported around 26% of total fat content.

Regarding the differences found among samples, it is remarkable to say as that, although no big differences were found among studied samples, only Moringa brownie presented the lowest amount of all the fatty acids, also having the lowest concentration of MUFA, PUFA, n‐3, and n‐6 in a 15% in comparison with the rest of studied samples. This fact can justify by the action of Moringa and their components to metabolize the fat.

### 3.4 Amino acid profile

**FIGURE 2:**
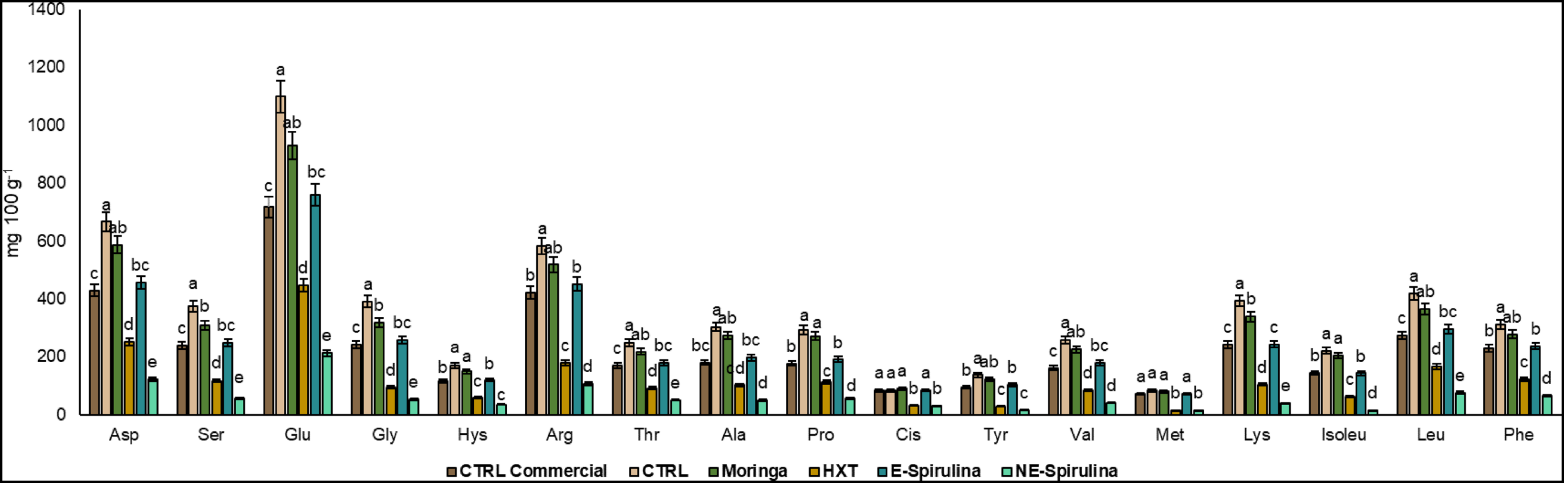
Profile of amino acids (mg 100 g^−1^) of studied formulas of brownies. Different letters denote significant differences among reformulated brownies (*p* < .05).

As shown in Figure 2, the most concentrated amino acid was glutamic acid (Glu), followed by aspartic acid (Asp), arginine (Arg), serine (Ser), glycine (Gly), lysine (Lys), leucine (Leu), phenylalanine (Phe), alanine (Ala), proline (Pro), threonine (Thr), valine (Val), isoleucine (Ile), histidine (His), tyrosine (Tyr), cysteine (Cis), and methionine (Met). When checked the levels of each compound, it is shown as Glu reaches values between 800 and 1200 mg per 100 g of brownie. In this sense, similar tendence has been previously found by Wu et al. (2002) who showed the highest values of Glu and the lowest values or Trp (does not detected in this study) in two kinds of brownies made of wheat or corn.

Moreover, it is remarkable that all the identified and quantified amino acids followed the same tendency for all the reformulated samples. In this sense, CTRL sample was always the richest followed by Moringa. E‐Spirulina and CTRL commercial did not show significant differences between them, and the lowest number of amino acids were found in those samples enriched in hydroxytyrosol (HXT) and, lastly, NE‐Spirulina.

This tendency shows as, between CTRL and CTRL commercial was always the first one the richest in amino acids, which indicates that was the quinoa flour instead of the maize flour the richest source in these nutrients (Arendt & Bello, 2008; Escuredo et al., 2014). Regarding those brownies which incorporated antioxidant compounds to their formula, Moringa has demonstrated to be also an important source of amino acids (Özcan, 2020; Witt, 2020), as well as Spirulina (Marti‐Quijal et al., 2019; Soni et al., 2017). Nevertheless, Spirulina and HXT are also important sources of phenolic compounds, which according to previous findings, could form conglomerates (cross‐linkings) with these amino acids to protect them against the thiol loss, and hence, the protein oxidation, which has been already demonstrated with catechins from tea (Jongberg et al., 2014) and phenolics from rosemary and oregano (Martínez, Jongberg, et al., 2020; Nieto et al., 2013). This theory could justify the reduction in the content of amino acids in the reformulated brownies with HXT and NE‐Spirulina. Therefore, this can be corroborated by the fact that the encapsulation of Spirulina is able to protect the phenols of this extract and to avoid their cross‐linking with the free amino acids of the brownies.

Furthermore, reformulated brownies in this study were an important source of essential amino acids, as His, Isoleu, Leu, Lys, Met, Phe, Thr, and Val, which must be incorporated to the body through the diet, as they are not synthesized by mammals.

### 3.5 Micronutrients

The results of the mineral determination are shown in Table 5. No Cr, K, Mo, Li, Ni, and Ti were found in the samples. As observed, no significant differences were found regarding the content of Al and Bi. From a general point of view, the richest mineral found in the elaborated brownies were Si and Na, followed by B, Al, P, Fe, Zn, Ca, Cu, Mg, Mn, Rb, S, Sr, and Bi. CTRL commercial was the richest brownie in B, Na, and Si, while CTRL was the richest sample in Ca, Cu, Fe, Mg, Mn, and P. Particularly, the reformulated samples rich in antioxidants were the richest samples in S and Zn, and Mg and P (with no differences with CTRL).

**TABLE 5:** Mineral content (mg 100 g^−1^) of reformulated brownies.

	CTRL commercial	CTRL	Moringa	HXT	E‐Spirulina	NE‐Spirulina
Al	28.5 ± 18.4	8.8 ± 0.3	28.7 ± 4.1	19.0 ± 7.7	9.6 ± 0.4	6.2 ± 0.3
Bi	0.6 ± 0.1	0.4 ± 0.1	0.6 ± 0.2	0.3 ± 0.1	0.6 ± 0.3	0.5 ± 0.0
B	75.4 ± 15.4A	12.0 ± 4.3C	13.7 ± 2.2C	27.3 ± 1.4B	14.6 ± 2.0C	11.6 ± 0.9C
Ca	5.0 ± 0.01A	3.5 ± 0.02B	4.5 ± 0.01AB	4.5 ± 0.01AB	4.5 ± 0.01AB	4.0 ± 0.01AB
Cu	2.0 ± 0.1A	0.8 ± 1.3B	1.3 ± 0.1AB	1.1 ± 0.1AB	1.3 ± 0.1AB	1.2 ± 0.1AB
Fe	13.2 ± 4.4A	8.4 ± 1.9AB	6.2 ± 2.0AB	5.6 ± 0.9AB	5.9 ± 0.1AB	4.7 ± 0.1B
Mg	1.0 ± 0.0B	3.0 ± 0.1A	3.0 ± 0.1A	2.5 ± 0.1A	3.0 ± 0.1A	2.5 ± 0.1A
Mn	2.0 ± 0.1C	4.3 ± 0.5A	4.1 ± 0.2AB	4.0 ± 0.2AB	4.4 ± 0.2A	3.7 ± 0.1B
Na	285 ± 6.0A	210 ± 10.0B	225 ± 10.0AB	245 ± 5.3AB	230 ± 2.1AB	215 ± 10.1B
P	8.5 ± 0.5B	15.5 ± 0.2A	15.0 ± 0.1A	14.5 ± 0.2A	15.5 ± 0.1A	14.0 ± 0.0A
Rb	2.1 ± 0.0C	2.4 ± 0.1AB	2.5 ± 0.1A	2.3 ± 0.0AB	2.4 ± 0.2AB	2.2 ± 0.0 BC
Si	712.6 ± 102.2A	151.4 ± 20.6B	176.4 ± 17.9B	360.9 ± 43.6AB	236.6 ± 56.2B	162.9 ± 14.9B
S	2.0 ± 0.0B	2.5 ± 0.0AB	3.0 ± 0.0A	3.0 ± 0.00A	3.0 ± 0.00A	3.0 ± 0.0A
Sr	0.4 ± 0.0C	0.7 ± 0.0B	1.2 ± 0.2A	0.9 ± 0.1B	0.9 ± 0.1B	0.8 ± 0.0B
Zn	3.4 ± 0.1B	4.9 ± 1.8B	9.0 ± 1.5A	8.1 ± 0.3A	8.7 ± 0.6A	7.7 ± 0.2A

*Note*: Different letters denote significant differences among reformulated brownies (*p* < .05).

**FIGURE 3:**
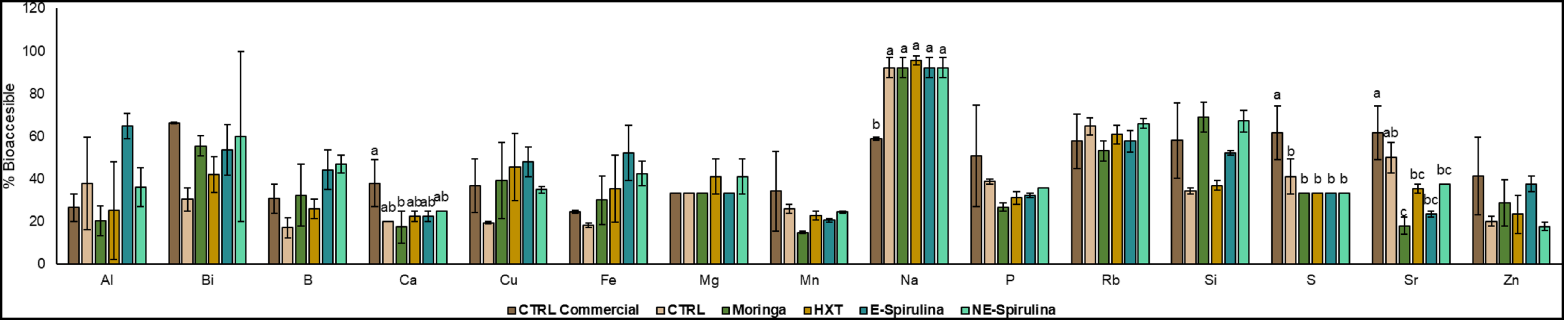
Percentage of bioaccessible minerals of studied formulas of brownies. Different letters denote significant differences among reformulated brownies (*p* < .05).

According to these data and the once obtained from analysis of digested formulas, the bioaccessible fraction (in percentage) was calculated and results are shown in Figure 3. As observed, the most bioaccessible mineral was Na, followed by Rb, Si, Bi, Al, Cu, S, Sr, Fe, Mg, P, Mn, B, Ca, and Zn. Although not so many differences were found among studied samples. In fact, CTRL commercial presented ~80% higher bioaccessibility of Ca, S, and Sr compared to the rest of reformulated brownies. By contrast, reformulated samples presented a higher (~40%) bioaccessibility of Na.

This fact confirms the hypothesis cited in the literature which relates the supplementation of functional foods with fiber to a decrease in the bioavailability of certain minerals, in particular, Ca, linked to certain fiber component, as phytates, which give rise to insoluble complexes such as Ca(OH)_2_ or Mg(OH)_2_ (Kiewlicz & Rybicka, 2020). Quinoa contains higher amounts of phytates than other pseudocereals, which would explain why all the brownies made experimentally showed a low bioavailability of these minerals (Ca, Cu, and Fe).

Also noteworthy is the increased Fe bioavailability (Figure 3) of brownies enriched with encapsulated and nonencapsulated spirulina compared to the other samples. These data are consistent with research on Fe bioavailability observed in mice after feeding with cultured, commercially available spirulina. The results showed that cultured spirulina was as good a source of Fe as FeSO_4_ (Johnson & Elliot Shubert, 1986). This research also pointed to the ability of spirulina to absorb large amounts of trace elements during growth, so that chronic use of spirulina could lead to intakes of elements such as Mg or lead above a safe level. However, subsequent research showed that toxic levels of both metals would require the ingestion of 77 g of spirulina daily, so its use as an extract for the fortification of bakery products is considered safe (Ekong et al., 2017).

Another study comparing the mineral and trace element content of three cereals of Andean origin (quinoa, amaranth, and purple corn) revealed high levels of Cu and Mn after ICP analysis of quinoa (Nascimento et al., 2014). These data are consistent with those obtained experimentally, with the exception of the bioavailability of Mn in the sample enriched with *Moringa oleifera*, in which a lower percentage of the element is observed than in the rest of the samples in which quinoa flour was used.

### 3.6 Bioactive compounds

**FIGURE 4:**
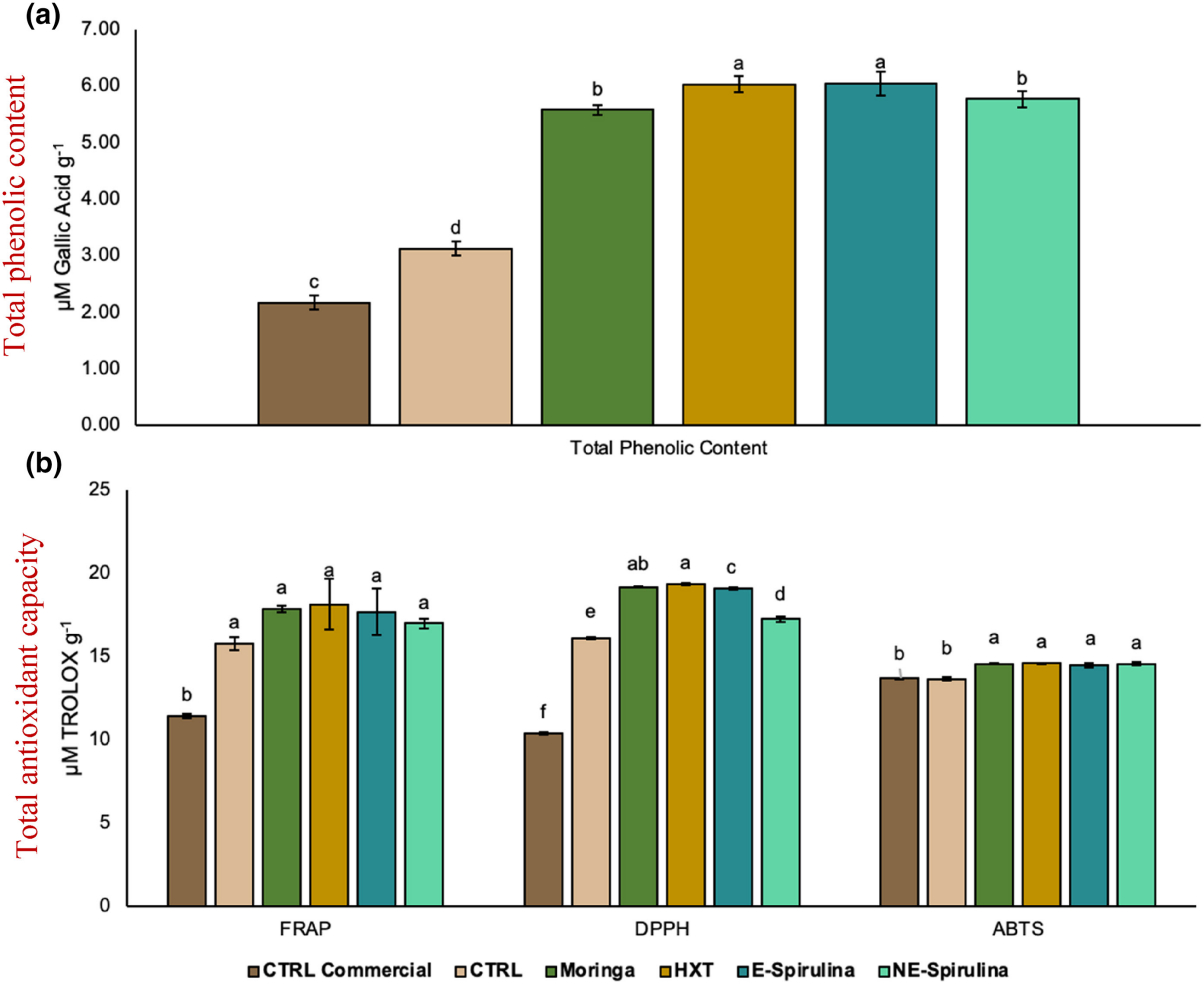
Total phenolic content (a) and total antioxidant capacity (b) of reformulated brownies. Different letters denote significant differences among reformulated brownies (*p* < .05).

Antioxidant activity has been determined by ABTS, DDPH, and FRAP tests while total phenolic compounds was evaluated by FOLIN test and obtained results are shown in Figure 4. Firstly, the results show that the brownies Moringa, HXT, E‐Spirulina, and NE‐Spirulina extracts show higher antioxidant activity and significantly higher total phenolic compounds (*p* < .05) than both CTRL and CTRL commercial.

In fact, these new formulas mainly increased by ~169% the total phenolic content compared to CTRL commercial and by ~87% compared to CTRL. When studied the total antioxidant capacity the behavior is similar, although each method reports different values of antioxidative action. For instance, the ability to reduce the iron (FRAP) was ~ 55% higher in reformulated formulas than CTRL commercial and ~12% higher than CTRL. The scavenging activity against DPPH free radical was also higher compared to CTRL commercial (~80%) than to CTRL (~16%). By contrast, the scavenging activity against ABTS free radical only was increased by ~6.5% in comparison with both CTRL samples.

In this sense, the increase of total phenolic content and antioxidant capacity of CTRL compared to CTRL commercial can be due to the presence of quinoa flour in its formula, which has already demonstrated its high antioxidant power compared to other pseudocereals such as Amaranth or rice (Arendt & Bello, 2008). In fact, also recently, Cannas et al. (2020)) showed the highly increased in the polyphenol fraction, flavonoids, and antioxidant activity after the substitution of rice flour by quinoa flour in gluten‐free ladyfinger biscuits. In fact, when these authors analyze the single flours, the results demonstrated an increase by 11‐fold in the polyphenol fraction and by twofold in the antioxidant activity of quinoa flour in comparison with rice flour.

Among the antioxidant compounds evaluated, the results obtained with phenylethanoid HXT, obtained as a by‐product in the manufacture of olive oil have been widely described (Martínez et al., 2018; Martínez‐Zamora et al., 2021). The antioxidant capacity of this compound has already been assessed in previous studies in which it has been found that a concentration of 100 ppm of HTX decreases lipid oxidation between 12% and 38% in fermented sausages (Chaves‐López et al., 2015). On the other hand, another study concluded that omega‐3 enrichment together with different amounts of HTX (100, 200, and 400 ppm) increased the antioxidant capacity in pies and caused a decrease in lipid oxidation on days 3, 6, and 9 (Muíño et al., 2017). More recently, HTX has demonstrated to be highly antioxidant compound in lamb patties (Martínez‐Zamora et al., 2020), chicken Nuggets (Martínez, Ros, & Nieto, 2020), and dry cured sausages (Zamora et al., 2021). In this sense, the high antioxidant capacity of this compound, despite its concentration being the lowest of all the extracts, can be explained by the presence of an OH group on its benzene ring, for which a higher function as a free radical scavenger, increasing its antioxidant power as well as its efficacy under stress conditions (Martínez et al., 2018; Martínez‐Zamora et al., 2021).

Another extract that has reported a notable antioxidant effect is *Moringa oleifera*. A study using this extract as a neuroprotective agent against the detrimental effects of Al absorption concluded that *Moringa oleifera* leaf extract reduced the serum concentration of Al, thus preventing it from reaching the temporal cortex (Ekong et al., 2017). This decrease in serum Al concentration could be the result of a chelation reaction between *Moringa oleifera* and the metal, as occurs with other antioxidants such as curcumin with the metal Cd (Ekong et al., 2017).

Additionally, also spirulina supplementation showed a high antioxidant activity comparable to HXT and Moringa, mainly when this microalga was encapsulated, while it reduces slightly its antioxidant activity and total phenolic content when was incorporated nonencapsulated. As shown by previous authors, the antioxidant and anti‐inflammatory activity of spirulina has been described by several authors (Kumar et al., 2022; Q. Wu et al., 2016), which demonstrated its efficiency and antioxidant in reformulated brownies. Furthermore, also Mohammadi et al. (2023)) has recently demonstrated as thanks to the encapsulation of algae hydrolysates in liposomal systems, the antioxidant activity of spirulina was efficiently maintained from harsh conditions providing structure stability, antioxidative capacity preservation, and enhanced antimicrobial properties of the hydrolysates.

### 3.7 Sensory analysis

In the sensory analysis, different parameters of the organoleptic quality of each brownie were evaluated and tasters were asked to rank the samples from highest to lowest according to their taste. A total of 20 people participated in the analysis, 65% of whom were women and 35% men. The average age was 29.6 years. Sixty‐nine percent of them expressed their liking for the product to be evaluated before the tasting, while 31% were indifferent to it.

**FIGURE 5:**
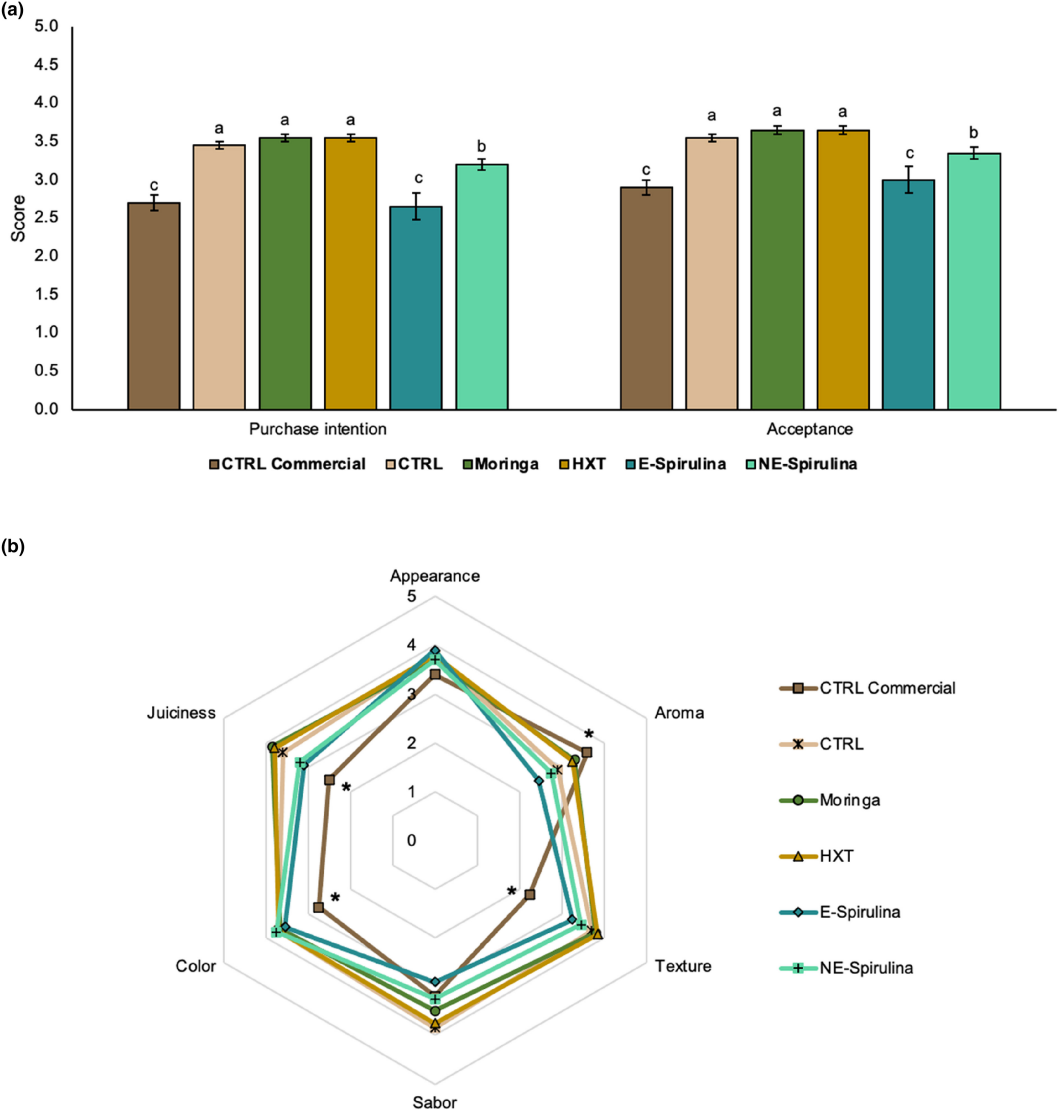
Acceptation (a) and sensory analysis (b) of reformulated brownies. Different letters in A and * in B denote significant differences among reformulated brownies (*p* < .05).

The samples with the best overall rating were the brownies Moringa and HXT with the same score, followed by CTRL and the NE‐Spirulina. The lowest score was obtained by the E‐Spirulina and the CTRL commercial with no significant differences among them (Figure 5a). In the parameters of appearance, taste, and color, there were no major differences between the samples (Figure 5b). It is worth noting the low values obtained for the aroma of the encapsulated and nonencapsulated spirulina samples (2.45 and 2.75, respectively). This may be attributed to the strong marine odor of this algae in populations not accustomed to its consumption (Dillon et al., 1995).

The CTRL commercial obtained values lower than 3 in texture, color, and purchase intention, being also the only sample that presented a value lower than 3 in the global acceptance of the product. In addition, it presented the lowest score in the evaluation of juiciness and texture, with significant differences (*p* < .05) with the rest of reformulated treatments. This fact, can due to the different flours used in the formulation.

As can be observed in the graph, the sample that obtained the highest frequency of first places was CTRL followed by Moringa, which is consistent with the previous results of the overall acceptance of the product. These data corroborate previous studies which conclude that the concentrations of *Moringa oleifera* used should not exceed 5%–10% to obtain sensory characteristics acceptable to consumers (Milla et al., 2021).

## 4 CONCLUSIONS

The brownies enriched in antioxidants and fiber fulfilled the starting hypothesis as they showed a higher antioxidant capacity and a substantial increase in the percentage of total dietary fiber, so they are postulated as a potential fortified bakery product. Following the development of a new brownie formulations with simultaneous psyllium and quinoa flour supplementation, an increase in the percentage of total fiber has been observed. However, the bioavailability of certain Ca or Fe nutrients has been reduced, so following the recommendations for the intake of high fiber foods, these minerals can be introduced in the diet in other moments of the day.

The evaluation of the different extracts reported significant elevations in antioxidant capacity and total phenolic compound composition, for which its regular role in the redox state of the cell as well as its preventive properties in the damage caused by oxidative stress it as a potential benefit to be consider in the prevention of pathologies such as cardiovascular or neurodegenerative diseases, diabetes or even cancer.

Although, all the extracts reported good results, Moringa showed lower values of some fatty acids and antioxidant activity, HXT the lower values of amino acids, and Spirulina the lower scores in the sensory analysis. Therefore, and in a balance of all the results and the low amount required for that, it will be recommendable the elaboration of fortified bakery products with HXT to enhance their antioxidant properties and sensory scores, together with quinoa flour and psyllium to increase the fiber content of this kind of products.

One limitation of this study would be the manufacture of this product in large quantities, as the incorporation of psyllium requires the addition of a precise amount of water in order to achieve a suitable texture for the dough with which to make the brownies. Therefore, the mass production of this product is possibly conditioned by the need for high precision in the choice of ingredient quantities.

## AUTHOR CONTRIBUTIONS

**Rocío Peñalver:** Formal analysis (lead); investigation (supporting); methodology (lead); writing – original draft (lead); writing – review and editing (supporting). **Lorena Martínez‐Zamora:** Investigation (supporting); writing – original draft (supporting). **Jose Manuel Lorenzo:** Software (equal). **Gaspar Ros:** Validation (supporting); visualization (supporting); writing – review and editing (supporting). **Gema Nieto Martínez:** Conceptualization (lead); data curation (lead); funding acquisition (lead); investigation (supporting); project administration (lead); supervision (lead); writing – original draft (supporting); writing – review and editing (lead).

## FUNDING INFORMATION

This work is part of the project PID2021‐123628OB‐C44, funded by MCIN/AEI/10.13039/501100011033.

## CONFLICT OF INTEREST STATEMENT

The authors declare that they have no conflicts of interest.

## Data Availability

Research data are not shared.
